# Optimising CT-guided biopsies of sclerotic bone lesions in cancer patients

**DOI:** 10.1007/s00330-022-09011-y

**Published:** 2022-07-26

**Authors:** Ricardo Donners, Nicos Fotiadis, Ines Figueiredo, Matthew Blackledge, Daniel Westaby, Christina Guo, Maria de los Dolores Fenor de la Maza, Dow-Mu Koh, Nina Tunariu

**Affiliations:** 1 Department of Radiology, Royal Marsden Hospital, Downs Road, Sutton, SM2 5PT London, UK; 2 Department of Radiology, University Hospital Basel, Petersgraben 4, 4031 Basel, Switzerland; 3 Department of Interventional Radiology, Royal Marsden Hospital, 203 Fulham Rd, London, SW3 6JJ UK; 4 The Institute of Cancer Research, 15 Cotswold Road, Sutton, SM2 5NG London, UK; 5 Cancer Research UK Cancer Imaging Centre, The Institute of Cancer Research, 15 Cotswold Road, Sutton, SM2 5NG London, UK

**Keywords:** Neoplasms, Image-guided biopsy, Computer tomography, Genomics, Bone marrow

## Abstract

**Objectives:**

Investigate the laboratory, imaging and procedural factors that are associated with a tumour-positive and/or NGS-feasible CT-guided sclerotic bone lesion biopsy result in cancer patients.

**Methods:**

In total, 113 CT-guided bone biopsies performed in cancer patients by an interventional radiologist in one institution were retrospectively reviewed. Sixty-five sclerotic bone biopsies were eventually included and routine blood parameters and tumour marker levels were recorded. Non-contrast (NC) biopsy CTs (65), contrast-enhanced CTs (24), and PET/CTs (22) performed within four weeks of biopsy were reviewed; lesion location, diameter, lesion-to-cortex distance, and NC-CT appearance (dense-sclerosis versus mild-sclerosis) were noted. Mean NC-CT, CE-CT HU, and PET SUVmax were derived from biopsy tract and lesion segmentations. Needle diameter, tract length, and number of samples were noted. Comparisons between tumour-positive/negative and next-generation sequencing (NGS)-feasible/non-feasible biopsies determined significant (*p* < 0.05) laboratory, imaging, and procedural parameter differences.

**Results:**

Seventy-four percent of biopsies were tumour-positive. NGS was feasible in 22/30 prostate cancer patients (73%). Neither laboratory blood parameters, PET/CT availability, size, nor lesion-to-cortex distance affected diagnostic yield or NGS feasibility (*p* > 0.298). Eighty-seven percent of mildly sclerotic bone (mean 244 HU) biopsies were positive compared with 56% in dense sclerosis (622 HU, *p* = 0.005) and NC-CT lesion HU was significantly lower in positive biopsies (*p* = 0.003). A 610 HU threshold yielded 89% PPV for tumour-positive biopsies and a 370 HU threshold 94% PPV for NGS-feasible biopsies. FDG-PET and procedural parameters were non-significant factors (each *p* > 0.055).

**Conclusion:**

In cancer patients with sclerotic bone disease, targeting areas of predominantly mild sclerosis in lower CT-attenuation lesions can improve tumour tissue yield and NGS feasibility.

**Key Points:**

*• Areas of predominantly mild sclerosis should be preferred to areas of predominantly dense sclerosis for CT-guided bone biopsies in cancer patients.*

*• Among sclerotic bone lesions in prostate cancer patients, lesions with a mean HU below 370 should be preferred as biopsy targets to improve NGS feasibility.*

*• Laboratory parameters and procedure related factors may have little implications for CT-guided sclerotic bone biopsy success.*

## Introduction

Tumour tissue sampling is becoming increasingly important in the age of personalised oncology [1, 2]. With dedicated therapies targeting unique tumour pathways, knowledge of specific tumour genomics is key towards patient selection. Consequently, access to high-quality tissue samples for advanced molecular analyses such as next-generation genomic sequencing (NGS) is paramount. Sclerotic bone lesions are often viewed as suboptimal biopsy targets, because of the high likelihood of tumour-negative results and return of only scant amounts of tumour cells, insufficient for NGS [3–6]. However, sclerotic bone lesions may be the only accessible manifestations in some malignancies, such as breast and prostate cancer, where up to 50% of patients present exclusively with skeletal disease [7–9].

A contemporary meta-analysis, including malignant and benign conditions, revealed a 74% success rate of CT-guided sclerotic bone lesion biopsies in providing sufficient yield for definitive histopathology diagnosis [3]. Lower tumour-positive rates between 50 and 53% were published for CT-guided bone biopsies performed in prostate cancer patients with sclerotic bone disease [6, 10]. Genomic sequencing feasibility rate was reported to be 45% for sclerotic bone biopsies in prostate cancer patients [11]. When including bone biopsies performed in lytic in addition to sclerotic bone lesions, higher sequencing success rates between 39 and 82% were described [10–12]. Generally, authors favour lytic (low CT-attenuation) over sclerotic (high CT-attenuation) lesions for CT-guided bone biopsies [4, 5, 11, 13, 14]. However, the former may be absent in some malignancies [9]. Therefore, further decision-support criteria for optimal biopsy target selection in cancer patients with exclusively sclerotic bone lesions are needed.

To optimise the CT-guided sclerotic bone biopsy workflow, maximise tumour tissue yield, and minimise tumour-negative and low-yield biopsies, the following are relevant considerations:

1. Patient selection—do laboratory blood parameters correlate with the likelihood of a tumour-positive bone biopsy result?
2. Lesion selection—which sclerotic bone lesion imaging features are associated with biopsy success?
3. Biopsy technique—do needle diameter, biopsy tract length, and number of cores taken per lesion impact CT-guided bone biopsy success?

The current literature provides conflicting information regarding the association of laboratory markers such as alkaline phosphatase (ALP) with a positive bone biopsy result [6, 15]. Targeting larger, progressing, and PET-positive bone lesions has been suggested to increase diagnostic yield [4, 5, 11, 13, 14]. With regards to bone biopsy procedure-related factors, most prior studies found little impact of needle diameter, sample size, number of cores obtained, and biopsy location on biopsy success [4–6, 10–13]. However, these features have not been evaluated in exclusively sclerotic bone lesions. Overall, recommendations for target selection in the sclerotic bone marrow are not established.

In this study, we investigate the laboratory, imaging, and procedural factors that are associated with a tumour-positive and NGS-feasible CT-guided sclerotic bone lesion biopsy result in cancer patients.

## Materials and methods

This retrospective single-centre study was conducted according to the legal regulations of the institutional review board. Requirement for informed patient consent was waived.

### Patients

**Fig. 1:**
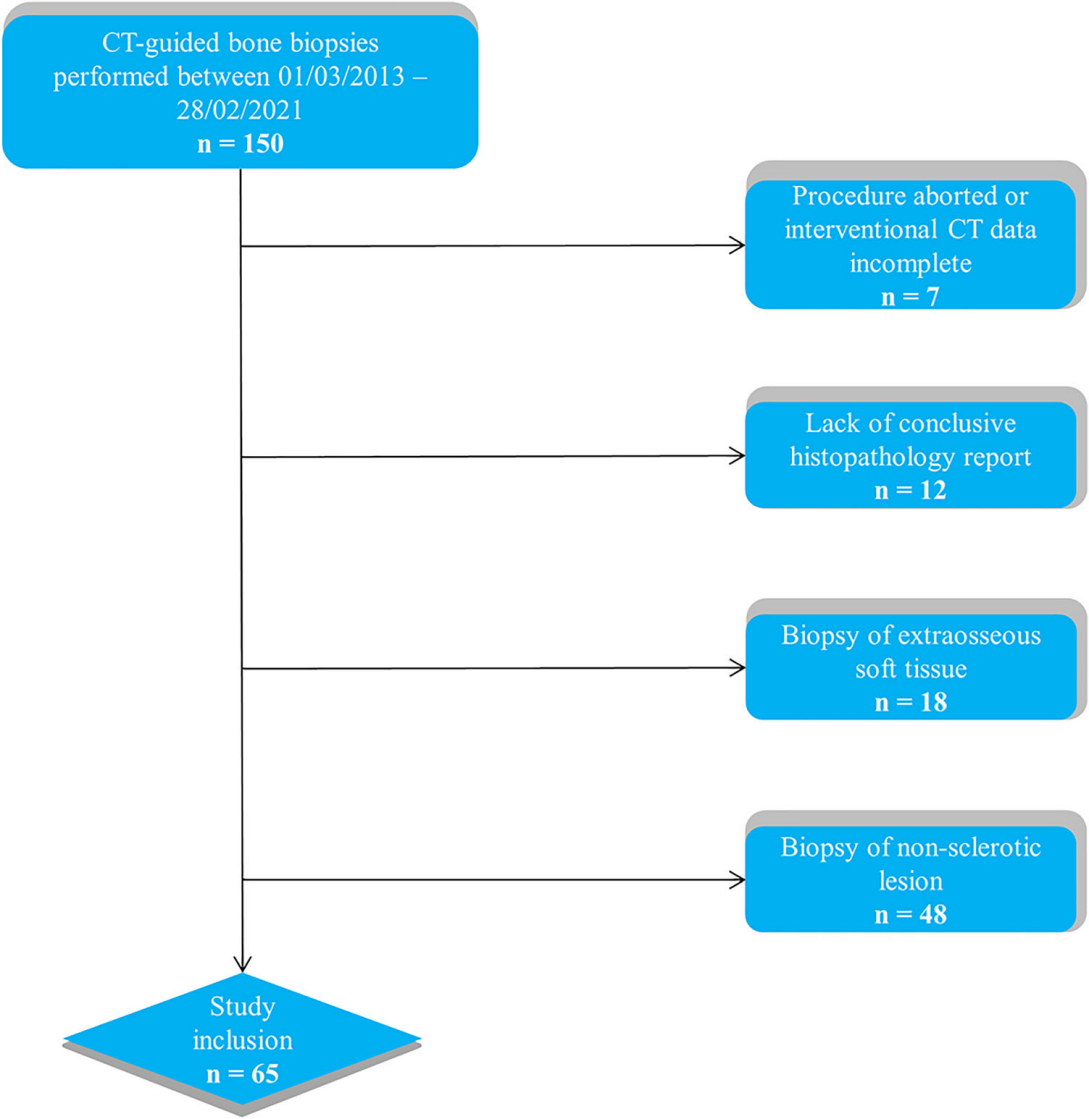
Study inclusion process, *n* = number of patients

Within our institution’s picture archiving and communication system (PACS, IDS7 Version 22.1, Sectra) a manual search for imaging studies labelled as “CT-guided bone biopsies” was performed. The search was limited to the time interval 01/03/2013 to 28/02/2021 and 150 unique imaging studies were identified. The recruitment process for this retrospective analysis is visualised in Figure 1. Inclusion criteria were as follows: CT-guided bone biopsy of a sclerotic bone lesion, the biopsy was performed in our department, the patients had a histopathologically established cancer diagnosis, availability of the biopsy CT (Bx-CT) and the histopathology bone biopsy report with a final tissue diagnosis. Patients were excluded when the procedure was aborted, interventional imaging was incomplete, conclusive histopathology results were lacking or when extraosseous soft tissue components of bone lesions were biopsied rather than the intraosseous marrow lesion. In total, 113 CT-guided bone biopsies with complete interventional imaging and histopathology reports were available. Among these, 36 biopsies were performed on lytic bone lesions and 12 lesions were not visible on CT, but were identified on previous MRI or PET/CT. After exclusion of these biopsies, ultimately, 65 CT-guided biopsies of sclerotic bone lesions, each performed on a unique patient, were included for final analysis in this study.

Patient demographics and blood parameters, including ALP in Units/L, lactate dehydrogenase (LDH) in Units/L, haemoglobin (Hb) in g/L, and platelets x10^9^/L obtained within 4 weeks prior to biopsy were noted. In prostate cancer patients, prostate-specific antigen (PSA) levels in μg/L and Gleason score were recorded.

### Image evaluation

A diagnostic and interventional radiology fellow with 3 years of experience in multimodality imaging of malignant bone disease, competent in performing CT-guided bone biopsies, evaluated all available imaging. Portal venous phase contrast-enhanced CT (CE-CT) and PET/CT studies performed within 4 weeks prior to the CT-guided bone biopsy were included for analysis. Imaging was assessed on commercially available software (OsiriX, version 56, PixmeoSARL Bernex). In review of all available imaging, maximum lesion diameter in biopsy direction and lesion-to-cortex distance in biopsy direction were recorded.

#### Biopsy tract and lesion segmentation

**Fig. 2:**
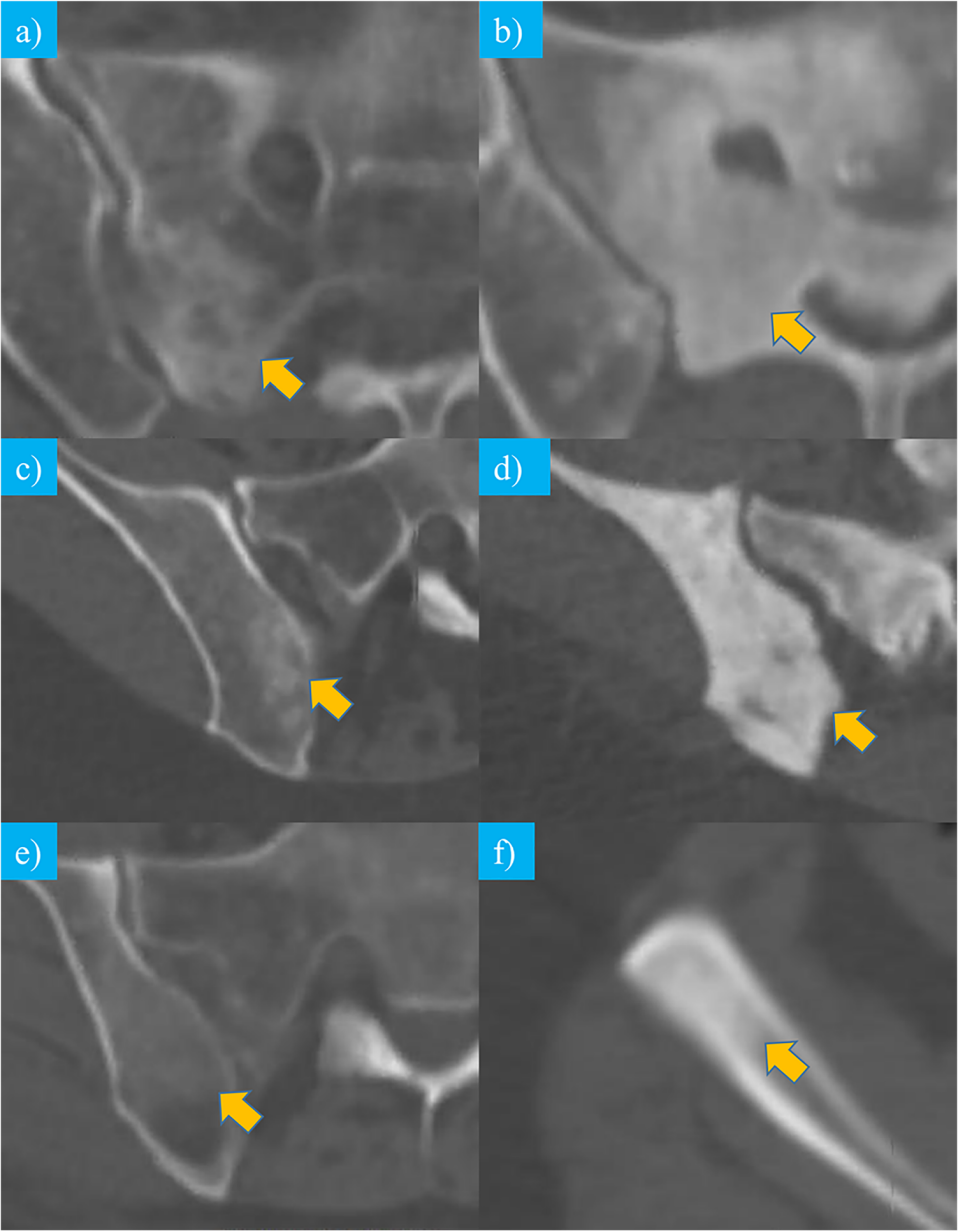
Types of lesion CT densities, mild sclerosis shown in **a**, **c**, and **e** in comparison to dense sclerosis in **b**, **d**, and **f**. **a** and **b** show breast cancer metastases, **c** and **d** prostate cancer metastases, and **e** and **f** bone marrow lymphoma

Segmentations of the biopsy tract and lesion were performed using OsiriX. On the Bx-CT images acquired immediately after the biopsy procedure, a volume of interest (VOI) was generated that included the intramedullary biopsy tract ± 3 mm of surrounding bone marrow. The VOI was transferred onto the corresponding pre-procedural non-contrast (NC) planning scan, which was performed immediately before the biopsy. On these NC-CT images, the CT density of the lesion intersected by the biopsy tract was visually assessed. NC-CT appearance was graded as predominantly dense sclerosis or predominantly mild sclerosis. The terms were used similarly to the visual grading system introduced by Holmes et al [6]. Lesion examples of predominantly dense and mild sclerosis are shown in Figure 2.

**Fig. 3:**
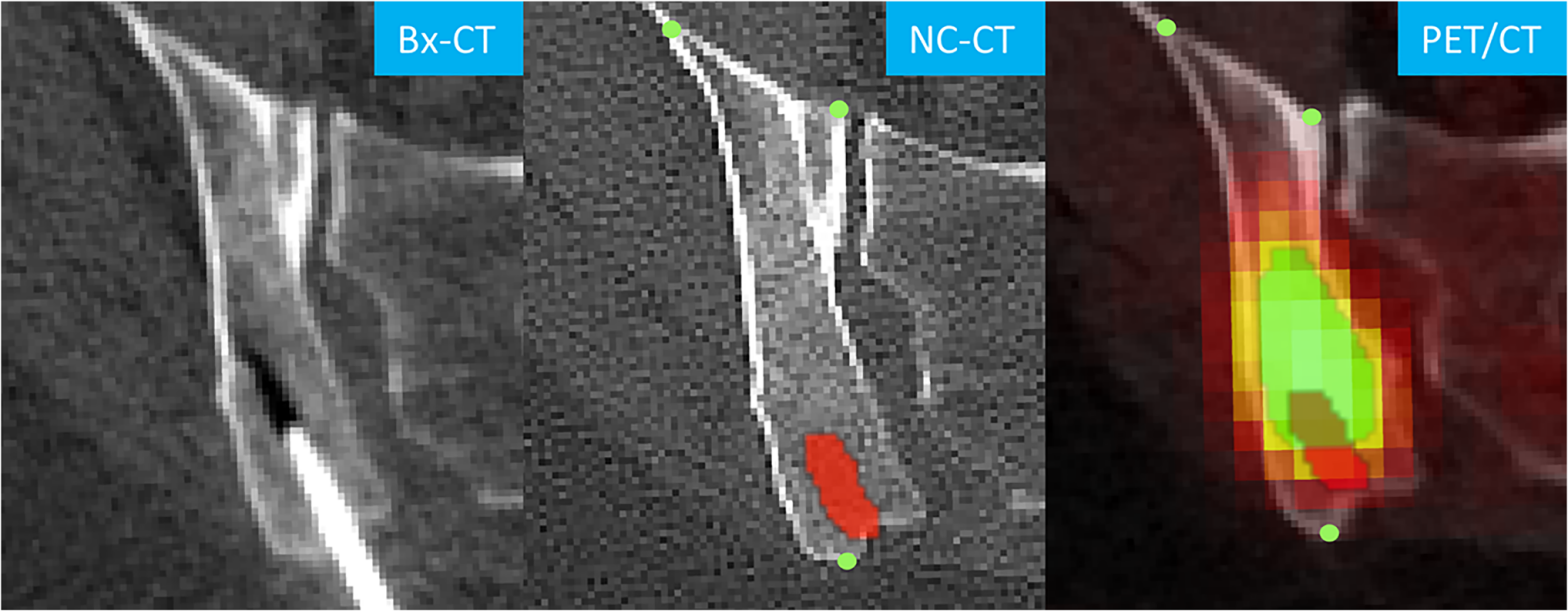
Right posterior iliac blade metastasis in a 67-year-old lymphoma patient. The biopsy-CT (Bx-CT) shows the biopsy needle in situ, the corresponding tract is delineated (red) on the non-contrast CT (NC-CT) and transferred onto the PET/CT using anatomical reference points (green dots, magnified for illustration purposes). The bone lesion was delineated on PET/CT (green) and is intersected by the biopsy tract

The generated biopsy tract VOI was transferred from the NC-CT onto the corresponding CE-CT and PET/CT images, using an in-house developed python®-based registration tool applied within OsiriX. The tool used anatomical reference points, which were placed manually on easily recognizable structures, such as the tip of the iliac spine, surrounding the biopsied lesion. This was performed for all modalities and allowed to copy the VOIs using the registration tool without altering the source images (Figure 3). The lack of spatial resolution of the PET images prevented the reliable identification of reference points. Thus, the CT component of the PET/CT was used for VOI transfer. VOI registrations were checked by the radiology fellow for coherence. Manual adjustment of the VOIs was not necessary in this study. The targeted lesion itself was delineated as visualised on NC-CT, CE-CT, and PET/CT, respectively. From the biopsy tract and target lesion VOIs, means of NC-CT Hounsfield (HU), CE-CT HU, and PET SUVmax were derived.

### Biopsy procedure

A specialised interventional radiologist, who had been performing CT-guided bone biopsies for 15 years or a supervised interventional radiology fellow, performed all biopsies included in this study. Target lesions were chosen based on the clinical judgement after review of all available CT, PET/CT, and/or MRI acquired prior to the procedure. Biopsies were performed under local anaesthesia and conscious sedation. Jamshidi™ (Becton, Dickinson and Company) or Madison™ (Merit Medical) bone biopsy needles were used.

During biopsies performed for NGS, a member of the histopathology team was present and instructed the interventional radiologist on the number of samples required as per trial protocol. For diagnostic biopsies multiple cores were only obtained, when the radiologist felt that the first sample was not representative or would likely provide insufficient material for a histopathology diagnosis.

The following procedure parameters were recorded for this study: gauge (G) of the biopsy needle, length of the biopsy tract, and number of bone samples obtained.

### Histopathology

After the biopsy cores were obtained, they were fixed for 24–30 h and decalcified in an EDTA solution for two days. Tumour content was assessed by a trained pathologist. Bone biopsy samples were deemed suitable for NGS when showing at least 150 cancer cells on H&E-stained, 2-μm-thick specimen slices. Ten-by-six-micrometre sections were prepared for sequencing. Whenever possible, a minimum of 20% tumour content was pursued by either coring or macro dissection following Nuclear Fast Red staining in cases where the tumour cells were not dispersed throughout the tissue sample. Biopsy specimens containing less than 150 tumour cells on H&E-stained slices were deemed not suitable for NGS by the pathologist.

### Statistical analyses

Statistical analyses were performed using commercially available software (IBM SPSS Statistics Version 25, IBM Corp.). Qualitative, nominal scaled variables were compared using chi-squared tests. Quantitative, continuous variables were compared using Mann-Whitney-U tests. A *p* value < 0.05 was deemed statistically significant. In cases of quantitative parameter significance, ROC AUC analyses were performed and optimised threshold values, discriminating between tumour-positive versus negative and NGS-feasible versus non-feasible biopsies were derived utilising the Youden Index.

## Results

Twenty female and 45 male patients with a median age of 67 years (range 34–82 years) were included. Patient demographics, tumour-positive, and NGS feasibility rates per primary malignancy are presented in Table 1. Histopathology diagnosis was malignant (tumour-positive) in 48 biopsy specimens (74%), normal or scarred bone marrow in seven (11%), deemed insufficient for diagnosis in seven (11%), and necrosis in three (4%) samples. NGS was only performed in prostate cancer patients and was feasible in 22/30 cases (73%). As such, the presented NGS feasibility results apply only to prostate cancer bone metastases.

**Table 1:** Patient characteristics and CT-guided bone biopsy results per primary malignancy. *NGS* next-generation genomic sequencing, *NA* not available, GIT - gastrointestinal tract

Primary malignancy	Median patient age (range)	Sex		Tumour positive	NGS feasible
		Female	Male		
Prostate cancer	67 (52–78)	0	37	27 (73%)	22/30 (73%)
Breast cancer	56 (34–72)	14	0	9 (64%)	NA
Lymphoma	66 (52–75)	4	3	7 (100%)	NA
Bronchial carcinoma	70 (68–71)	0	2	2 (100%)	NA
Mucinous adenocarcinoma of the GIT	71 (68–82)	2	1	2 (50%)	NA
Transitional cell carcinoma	66 (59–72)	0	2	1 (50%)	NA

### Laboratory blood parameters

LDH was the only laboratory parameter that showed a statistically significant correlation with a tumour-positive sclerotic bone biopsy result in this study. Significantly higher LDH levels were found in patients with tumour-positive (246 U/L) compared with negative biopsies (150 U/L, *p* = 0.048). 141 U/L was identified as an optimised threshold value, larger LDH values identifying positive biopsies with 81% sensitivity, 56% specificity, and 83% positive predictive value (PPV). Mean LDH was not significantly different between NGS feasible (270 U/L) and non-feasible (227 U/L, *p* = 0.959) biopsies.

Neither ALP, Hb, platelet count, PSA, nor Gleason score across all biopsies showed a significant correlation with a tumour-positive result. In prostate cancer, neither ALP, Hb, platelet count, PSA, nor Gleason score showed a significant correlation with NGS feasibility (each *p* > 0.298).

### PET/CT availability

FDG-PET/CT was available in 22 (11 breast adenocarcinoma, 5 lymphoma, 3 mucinous gastrointestinal cancer, 2 prostate adenocarcinoma, 1 transitional cell cancer), PSMA-PET/CT in two (prostate adenocarcinoma), and Choline-PET/CT in three (prostate adenocarcinoma) patients within four months prior to biopsy. There was no significant correlation between PET/CT availability and a tumour-positive or NGS-feasible biopsy result (each *p* > 0.164).

### Lesion size and lesion-to-cortex distance

The maximum lesion diameter in the biopsy direction was 26 ± 15 mm in tumour-positive and 25 ± 12 mm in negative biopsies (*p* = 0.914). The average cortex-to-lesion distance was 5 ± 8 mm in positive and 3 ± 5 mm in negative biopsies (*p* = 0.358). There was no significant difference in maximum lesion diameter nor lesion-to-cortex distance with regard to NGS feasibility in prostate cancer patients (each *p* > 0.723).

### CT appearance

**Fig. 4:**
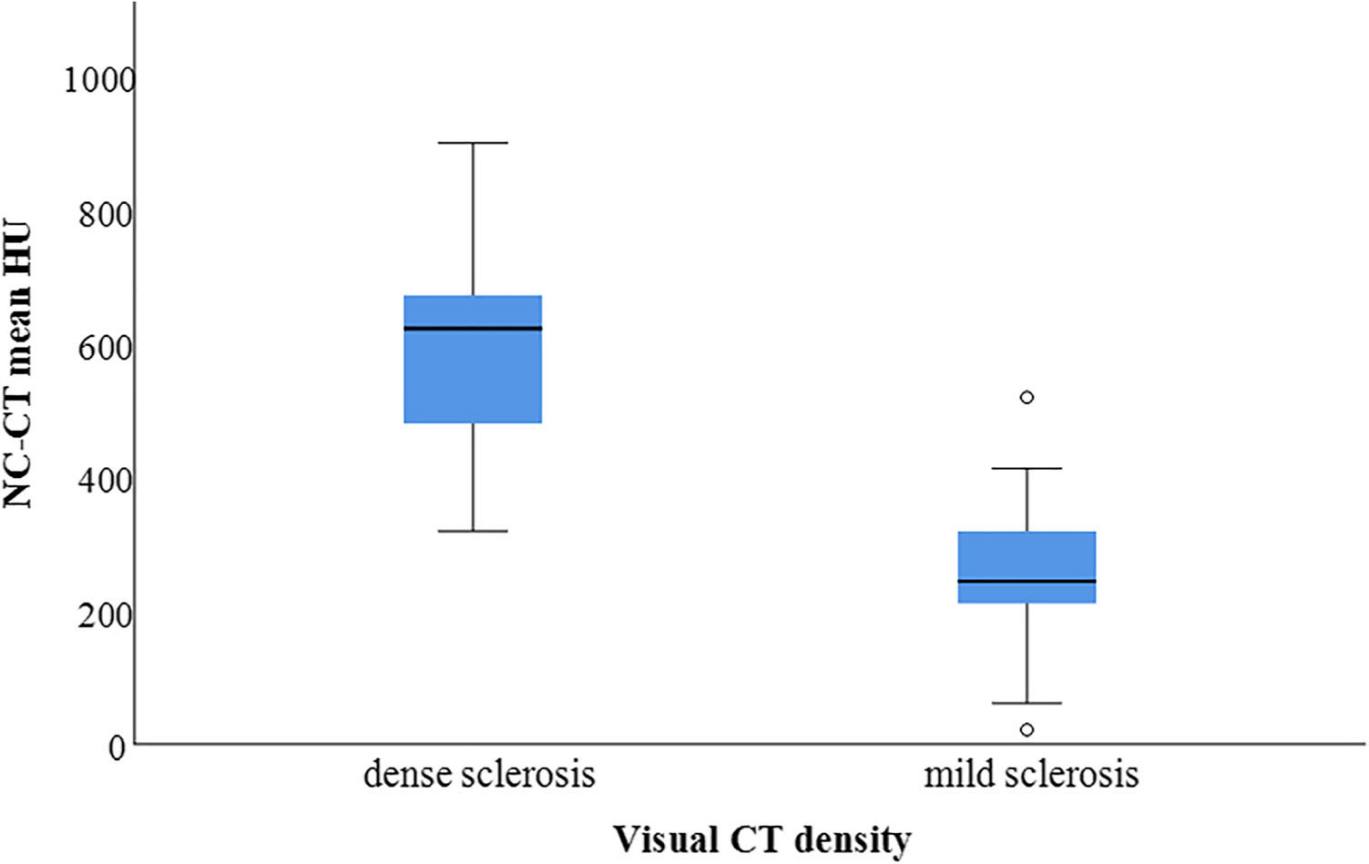
Box plot comparing the mean non-contrast CT HU (NC-CT) values between areas of predominantly dense and mild sclerosis

Table 2 summarises the biopsy results depending on the visual grading as predominantly dense or mild sclerosis. Mean HU ranges are visualised in Figure 4. Biopsies of predominantly mild sclerosis had a significantly greater tumour-positive rate compared with biopsies of predominantly dense sclerosis (*p* = 0.005). There was no significant difference regarding NGS feasibility (*p* = 0.077).

**Table 2:** CT-guided sclerotic bone biopsy results per CT appearance. *NGS* – next-generation genomic sequencing, ranges in squared brackets. *NGS* results apply only to prostate cancer metastases, biopsies of mild sclerosis had a significantly greater tumour-positive rate compared with biopsies of dense sclerosis (*p* = 0.005)

		Tumour positive		NGS	
CT appearance	Median HU	Yes	No	Feasible	Not feasible
**Dense sclerosis**	622 [319–900]	15 (56%)	12	6 (55%)	5
**Mild sclerosis**	244 [21–519]	33 (87%)	5	16 (84%)	3

### Quantitative imaging parameters

The average volume of the biopsy tract VOIs was 0.33 mL for NC-CT, 0.38 ml for CE-CT, and 0.29 ml for PET images. The average volume of lesion VOIs was 1.65 ml for NC-CT, 1.64 ml for CE-CT and 1.11 ml for PET images.

#### Biopsy tract–derived quantitative imaging parameters

There were no statistical differences for the quantitative biopsy tract parameters mean NC-CT HU, CT-CT HU and FDG/PET SUVmax between tumour-positive and negative nor between NGS-feasible and non-feasible biopsy results (each *p* > 0.160).

#### Lesion-derived quantitative imaging parameters

Table 3 summarises the derived measurements. Lesion analysis revealed significantly lower mean NC-CT HU values in tumour-positive compared with negative biopsies (*p* = 0.003). In total, 610 mean NC-CT HU was determined as the optimal threshold value. Lesions with < 610 mean NC-CT HU resulted in a significantly greater proportion of tumour-positive biopsies compared with > 610 mean NC/CT HU lesions. The 610 HU threshold yielded 84% sensitivity, 67% specificity, and 89% PPV for the identification of tumour-positive biopsies.

**Table 3:** Non-contrast CT (B-CT), contrast-enhanced CT (CE-CT), and FDG-PET/CT SUVmax measurements from biopsied lesion segmentation with a tumour-positive and tumour-negative biopsy result and next-generation genomic sequencing feasibility or failure. *SD* standard deviation

	Tumour				Next-generation genomic sequencing			
	Positive	Negative			Feasible	Not feasible		
	Mean *± SD*	Mean *± SD*	*p* value	*n*	Mean *± SD*	Mean *± SD*	*p* value	*n*
NC-CT HU	367 *± 191*	601 *± 215*	0.003	65	325 *± 192*	549 *± 183*	0.006	30
CE-CT HU	400 *± 216*	535 *± 204*	0.206	20	324 *± 287*	518 *± 171*	0.109	12
FDG/PET SUVmax	6.5 *± 5.0*	4.4 *± 2.5*	0.322	23				

Next-generation genomic sequencing (NGS) was only performed in prostate cancer patients, SUVmax values are missing as FDG-PET/CT was not available in these

Lesion analysis also showed significantly lower mean NC-CT HU values in NGS-feasible compared with NGS non-feasible biopsies (*p* = 0.006) in prostate cancer. In total, 370 mean NC-CT HU was calculated as the optimal threshold value. Biopsies of lesions with < 370 mean NC-CT HU showed a significantly higher NGS feasibility rate compared with biopsies in lesions measuring > 370 mean NC-CT HU. The 370 mean NC-CT HU threshold yielded 73% sensitivity, 88% specificity, and 94% PPV for the identification of NGS-feasible biopsies.

### Biopsy procedure parameters

Table 4 shows the procedure-related parameter comparison. There were no statistically significant differences when comparing the length of the biopsy tract, the number of cores obtained, or biopsy needle diameter between tumour-positive and negative biopsies (each *p* > 0.055). Likewise, no significant differences were found for these parameters between NGS feasible and non-feasible prostate cancer bone biopsies (each *p* > 0.060).

**Table 4:** Impact of biopsy procedure related factors on CT-guided bone biopsy results. Next-generation genomic sequencing results apply only to prostate cancer metastases

	Tumour positive			Next-generation genomic sequencing		
Parameter	Yes	No	*p* value	Feasible	Not feasible	*p* value
Mean biopsy needle Gauge	12 ± 1	12 ± 1	0.978	12.5 ± 2	12 ± 1	0.723
Mean tract length in mm	16 ± 8	12 ± 6	0.055	15 ± 7	10 ± 4	0.060
Mean number of cores obtained	2.3 ± 1.2	2.2 ± 1.4	0.585	3.2 ± 0.9	3.1 ± 1.5	0.656

### Optimised CT-guided sclerotic bone lesion biopsy

Table 5 summarises the performance of the statistically significant laboratory and imaging parameters associated with a tumour-positive and NGS-feasible CT-guided sclerotic bone biopsy result.

**Table 5:** Performance of laboratory and imaging parameters associated with biopsy success, Next-generation genomic sequencing results apply only to prostate cancer metastases

**Parameter**	PPV in %	NPV in %	Sensitivity in %	Specificity in %	PLR in %	NLR in %
**Tumour positive biopsy result**						
LDH > 141 U/L	83 (74–90)	53 (34–71)	81 (67–92)	56 (30–80)	1.9 (1.1–3.3)	0.3 (0.2–0.7)
Mild sclerosis bone biopsy*	87 (76–93)	44 (32–57)	69 (54–81)	71 (44–90)	2.3 (1.1–5.0)	0.4 (0.3–0.7)
610 HU NC-CT threshold	89 (77–95)	56 (36–75)	84 (68–94)	67 (35–90)	2.5 (1.1–5.7)	0.2 (0.1–0.6)
**Next-generation genomic sequencing feasibility**						
370 HU NC-CT threshold	94 (71–99)	54 (36–71)	73 (80–89)	88 (47–100)	5.8 (0.9–37.1)	0.3 (0.2–0.7)

*PPV* positive predictive value, *NPV* negative predictive value, *PLR* positive likelihood ration, *NPR* negative likelihood ratio, *LDH* lactate dehydrogenase, *NC-CT* non-contrast CT. 95% confidence intervals in brackets

## Discussion

In this study, we found that in cancer patients, CT-guided bone biopsies of predominantly mild sclerosis and lower CT attenuation lesions result in significantly greater tumour-positive and NGS feasibility rate, when compared with biopsies of dense sclerosis and higher attenuation lesions. Regarding a tumour-positive biopsy outcome, we found 610 HU as an optimal threshold with a PPV of 89% for lesions with smaller mean HU. With regards to NGS feasibility in prostate cancer patients, the optimised threshold was 370 HU, resulting in 94% PPV for lesions with smaller mean HU. By contrast, most laboratory or procedure-related parameters had no significant impact on the biopsy results.

LDH was the only laboratory blood parameter that showed a statistical significant correlation with the bone biopsy outcome, with higher LDH levels found in cancer patients with a positive bone biopsy. Although LDH is an established parameter for monitoring malignant disease [16], linking a systemic blood parameter to the success of local tissue sampling is questionable. Overall, the lack of correlation between the remaining blood parameter levels and bone biopsy results is in keeping with earlier observations [15]. Thus, systemic laboratory measurements may have limited implications for individual bone lesions.

Neither lesion size nor lesion-to-cortex distance differed significantly between tumour-positive and negative nor NGS feasible and non-feasible biopsies. This is in keeping with the published literature [6, 11].

Targeting lytic bone lesions for CT-guided bone biopsies is established as a general recommendation [4–6, 10, 13–15, 17]. However, in some patients, lytic disease is absent and sclerotic bone lesions are the only available biopsy targets. For these patients, target selection support criteria are required. Validating previous researchers, we found that areas of visually mild sclerosis should be favoured as biopsy targets over the densely sclerotic bone to improve tumour-tissue yield [6]. This subjective finding is supported by our quantitative analysis, as significantly lower lesion NC-CT HU values were found for successful biopsies. We identified 610 HU as the optimal threshold for identification of sclerotic bone lesions with a tumour-positive bone biopsy result, with lower values indicating bone disease suitable for biopsy. In prostate cancer patients, 370 HU was identified as the optimised threshold for the identification of bone biopsy targets resulting in NGS feasible results in our cohort. The discrepancy between the 610 HU diagnostic and 370 HU NGS threshold may be explained by the fact that significantly more tumour tissue is required for NGS compared with a histopathological tumour diagnosis. It can be hypothesised that mildly sclerotic, lower HU lesions contain more viable tumour than sclerotic, higher CT-attenuation bone marrow. Previously defined thresholds for the identification of tumour-positive biopsies range between 400 and 500 HU [6, 10, 18]. Improved tumour-positive bone biopsy rates of 70–79% in lower HU lesions compared with higher CT-attenuation lesions (33–40%) were also described [6, 18]. These thresholds included lytic or mixed-lytic lesions, while our study exclusively focussed on sclerotic bone, which may account for the HU difference. Optimised HU thresholds focussed on NGS feasibility from sclerotic bone lesion biopsies were not previously published.

Within the available data, differences in procedure-related parameters, including needle diameter, biopsy tract length, and number of core samples taken were not associated with a tumour-positive or NGS feasible result. Similar findings were made by previous researchers, who analysed the role of needle diameter, lesion-to-cortex distance, skin-to-lesion distance, tract length, and number of cores obtained [6, 10–12].

The overall tumour-positive rate of 74% in our study is within the pooled range of 62–82% and equal to the mean calculated across 13 studies analysed in a contemporary meta-analysis of CT-guided sclerotic bone biopsies [3]. The 73% NGS feasibility rate is within the range of previously published results, which included lytic and sclerotic bone lesions [6, 11, 12, 15, 17]. This should allow for the general applicability of our findings outside of our own institution, to improve CT-guided bone biopsy success rates in cancer patients with sclerotic bone disease. Although the threshold values need further validation in larger cohorts, our results suggest that for diagnostic biopsies bone lesions with mean values < 610 HU and for NGS in prostate cancer lesions < 370 HU should be targeted.

Our study has limitations. First, in this small, retrospective and single-centre cohort, all lesions had already undergone a selection process by the executing interventional radiologist, creating inclusion bias. Second, there was no histopathologic prove that negative biopsies targeted malignant lesions. Imaging suggested malignancy, and this study deliberately aimed to identify parameters that discriminate tumour-positive from tumour-negative and NGS feasible from non-feasible biopsies. Third, only a limited variety of bone biopsy needles are used in our department. Hence, the full scope of the hardware influence, including different biopsy systems and a wide range of needle diameters, on biopsy outcome cannot be evaluated. Moreover, the impact of electrical powered drills, which were suggested to give superior results was not assessed [3]. Fourth, NGS was only performed in prostate cancer patients. Thus, results can only be applied to this patient group. Moreover, the number of obtained samples was significantly higher for NGS than diagnostic biopsies, as patients were included in multiple trials, requiring separate specimens. Finally, PET/CT and especially PSMA PET/CT, which was previously shown to improve bone biopsy success in prostate cancer patients [15, 17], were available only in a small number of patients. As such, the presented study likely underestimates the value of PET/CT for bone biopsy target selection.

Concluding, in cancer patients with sclerotic bone disease, targeting areas of predominantly mild sclerosis in lower CT-attenuation lesions can improve tumour tissue yield and NGS feasibility. NC-CT sclerotic bone target lesion mean HU should preferably be less than 610 HU for diagnostic biopsies and less than 370 HU when biopsies are performed for NGS in prostate cancer patients.

## Abbreviations

Bx-CT: Biopsy CT
FO: Fat-only
GIT: Gastrointestinal tract
mpBRMI: Multiparametric bone MRI
NA: Not available
PSMA: Prostate-specific membrane antigen
PV: Portal venous
rFF: Relative fat fraction

## Declarations

### Guarantor

The scientific guarantor of this publication is Nina Tunariu.

### Statistics and biometry

No complex statistical methods were necessary for this paper.

### Study subjects or cohorts overlap

Twenty-nine of the included study subjects were previously reported in the recently published study "Multiparametric bone MRI can improve CT-guided bone biopsy target selection in cancer patients and increase diagnostic yield and feasibility of next-generation tumour sequencing". In this previous study, only MRI characteristics of these lesions were analysed irrespective of laboratory, technical and CT or PET/CT imaging features. In contrast, the submitted manuscript contains information on the latter, while not considering any MRI features.

### Methodology

• retrospective

• observational

• performed at one institution

## References

[1] Mukherjee S (2019). Genomics-guided immunotherapy for precision medicine in cancer. Cancer Biother Radiopharm..

[2] Smits M, Mehra N, Sedelaar M, Gerritsen W, Schalken JA (2017). Molecular biomarkers to guide precision medicine in localized prostate cancer. Expert Rev Mol Diagn..

[3] Suh CH, Yun SJ (2019). Diagnostic outcome of image-guided percutaneous core needle biopsy of sclerotic bone lesions: a meta-analysis. AJR Am J Roentgenol..

[4] Li Y, Du Y, Luo TY (2014). Factors influencing diagnostic yield of CT-guided percutaneous core needle biopsy for bone lesions. Clin Radiol..

[5] Wu JS, Goldsmith JD, Horwich PJ, Shetty SK, Hochman MG (2008). Bone and soft-tissue lesions: what factors affect diagnostic yield of image-guided core-needle biopsy?. Radiology.

[6] Holmes MG, Foss E, Joseph G, et al (2017) CT-guided bone biopsies in metastatic castration-resistant prostate cancer: factors predictive of maximum tumor yield. J Vasc Interv Radiol. 28(8):1073-81.e1. 10.1016/j.jvir.2017.04.019 28549709

[7] Coleman RE (2001). Metastatic bone disease: clinical features, pathophysiology and treatment strategies. Cancer Treat Rev..

[8] Coleman RE, Rubens RD (1987). The clinical course of bone metastases from breast cancer. Br J Cancer..

[9] Coleman RE, Croucher PI, Padhani AR (2020). Bone metastases. Nat Rev Dis Primers..

[10] McKay RR, Zukotynski KA, Werner L (2014). Imaging, procedural and clinical variables associated with tumor yield on bone biopsy in metastatic castration-resistant prostate cancer. Prostate Cancer Prostatic Dis..

[11] Spritzer CE, Afonso PD, Vinson EN (2013). Bone marrow biopsy: RNA isolation with expression profiling in men with metastatic castration-resistant prostate cancer--factors affecting diagnostic success. Radiology.

[12] Sailer V, Schiffman MH, Kossai M (2018). Bone biopsy protocol for advanced prostate cancer in the era of precision medicine. Cancer.

[13] Hao DJ, Sun HH, He BR, Liu TJ, Jiang YH, Zhao QP (2011). Accuracy of CT-guided biopsies in 158 patients with thoracic spinal lesions. Acta Radiol..

[14] Hwang S, Lefkowitz RA, Landa J (2011). Percutaneous CT-guided bone biopsy: diagnosis of malignancy in lesions with initially indeterminate biopsy results and CT features associated with diagnostic or indeterminate results. AJR Am J Roentgenol..

[15] de Jong AC, Smits M, van Riet J (2020). Ga-PSMA-guided bone biopsies for molecular diagnostics in patients with metastatic prostate cancer. J Nucl Med..

[16] Brown JE, Cook RJ, Lipton A, Coleman RE (2012). Serum lactate dehydrogenase is prognostic for survival in patients with bone metastases from breast cancer: a retrospective analysis in bisphosphonate-treated patients. Clin Cancer Res..

[17] Smits M, Ekici K, Pamidimarri Naga S et al (2020) Prior PSMA PET-CT imaging and Hounsfield unit impact on tumor yield and success of molecular analyses from bone biopsies in metastatic prostate cancer. Cancers (Basel) 12(12) 10.3390/cancers12123756 PMC7764855 33327413

[18] Ní Mhuircheartaigh J, McMahon C, Lin YC, Wu J (2017). Diagnostic yield of percutaneous biopsy for sclerotic bone lesions: Influence of mean Hounsfield units. Clin Imaging..

